# Comprehensive assessments of the open mouth dynamic maneuver and metal artifact reduction algorithm on computed tomography images of the oral cavity and oropharynx

**DOI:** 10.1371/journal.pone.0248696

**Published:** 2021-03-18

**Authors:** Yun Jung Bae, Tae Eun Kim, Byung Se Choi, Woo-Jin Jeong, Se Jin Cho, Sung Hyun Baik, Leonard Sunwoo, Jae Hyoung Kim

**Affiliations:** 1 Department of Radiology, Seoul National University Bundang Hospital, Seongnam, Republic of Korea; 2 Department of Otolaryngology–Head & Neck Surgery, Seoul National University Bundang Hospital, Seongnam, Republic of Korea; University of California, San Francisco, UNITED STATES

## Abstract

**Objectives:**

To determine the optimal utility of the open mouth maneuver and Metal Artifact Reduction for the Orthopedic Implants (O-MAR) technique for CT of the oral cavity and oropharynx.

**Methods:**

Between July 2017 and May 2019, 59 subjects who underwent both conventional and open mouth head and neck CT scans were included in this retrospective study. All images were reconstructed using the O-MAR algorithm. With conventional CT with/without the O-MAR (CTc_O/CTc) and open mouth CT with/without O-MAR (CTo_O/CTo), one reader measured the noise level in multiple anatomic regions of the oral cavity and oropharynx. Visual scores for the streak artifact and overall subjective image quality were assessed by two independent readers.

**Results:**

For the mobile tongue, retromolar trigone, and palatine tonsil, the mean noise was significantly lower, and the mean visual scores were significantly higher, with CTo than with CTc or CTc_O (all, *P* < 0.001). The mean visual scores were higher with CTo_O than with CTo for the mobile tongue and palatine tonsil (all, *P* < 0.001). Contrarily, for the mouth floor and tongue base, the mean noise was significantly higher with CTo_O than with CTc or CTc_O, and the mean visual scores were significantly higher with CTc than with CTo or CTo_O (all, *P* < 0.001).

**Conclusions:**

The open mouth maneuver and O-MAR technique can have different influences on the CT image quality according to the anatomical subsites of the oral cavity and oropharynx.

## Introduction

Computed tomography (CT) is a commonly used imaging tool for the staging and surveillance of patients with oral cavity and oropharyngeal cancer [1]. Cross-sectional imaging plays an important role in primary tumor staging, providing information regarding the size and possible invasion of deep anatomical structures such as the cortical bone or extrinsic tongue muscles [2]. CT is widely available with a low cost and short scan time, whereas magnetic resonance imaging (MRI) has limited availability, a high cost, and a longer scan time, which makes images vulnerable to movement due to swallowing and respiration [1].

Nevertheless, CT images of the oral cavity and oropharynx can be degraded due to metallic artifacts caused by dental hardware, thus hampering its clinical usefulness [1, 3, 4]. Dental hardware can produce photon starvation and beam hardening, which can obscure the primary tumor in the oral cavity, and the streak artifact from the prosthesis may cover the structures in the adjacent oropharynx [1, 3, 4]. To avoid such artifacts, a dynamic maneuver called “open mouth” has been described to improve tumor delineation by stretching the oral mucosa and the deep spaces in the vicinity [5, 6]. Most recently, Bron et al. [7] found that post-contrast CT images using the open mouth maneuver could improve the tumor delineation and decrease metallic artifacts in the oral cavity and oropharynx, thereby increasing the clinical advantages for proper tumor staging, comparable to MRI examination.

Another method to reduce metallic artifacts is the well-known “Metal Artifact Reduction (MAR)” technique, which is a post-processing algorithm for mitigating metallic artifacts using an iterative reconstruction method [8]. Previous studies showed that the application of MAR on CT can better depict structures in the oro-maxillary region, which are not visible on uncorrected images with metallic artifacts [1, 3, 4, 8, 9]. However, this method can generate unwanted effects such as blurring and unnatural texture, which remains a main technical challenge for its clinical utilization [1, 3, 4, 8, 9].

In our study, we aimed to perform a comprehensive assessment of head and neck CT image quality using different metallic artifact reduction techniques. To the best of our knowledge, combined consideration of the open mouth maneuver and MAR technique on head and neck CT image quality has not yet been investigated. We applied each intervention alone and in combination on the CT images of the oral cavity and oropharynx and evaluated the objective and the subjective image quality of the detailed anatomical subsites. The purpose of our study was to determine the optimal utility of the open mouth maneuver and MAR technique for the oral cavity and oropharynx on CT images.

## Materials and methods

### Subjects

This retrospective study was approved by the Institutional Review Board of Seoul National University Bundang Hospital (B-2001-592-106), and the requirement for written informed consent was waived. Between July 2017 and May 2019, 381 subjects underwent head and neck CT imaging targeting the oral cavity and oropharynx for various clinical purposes. Among them, for those who underwent CT scans in the scanners with installed MAR algorithm and those who had dental prosthesis producing a metallic artifact that obscured the anatomical subsites of the oral cavity and oropharynx, an additional CT scan using the open mouth maneuver was performed for evaluating the oral cavity and oropharynx. Subjects under the age of 20 (n = 31) did not undergo the additional CT scan. In total, 59 subjects (31 men and 28 women; age range, 26–81 years; mean age, 58.7 years) who had both conventional and open mouth CT scans with available MAR application were included in the study. The underlying pathologies or chief symptoms of the included subjects were as follows: branchial cleft cyst (n = 2), burning mouth syndrome (n = 2), dysphagia (n = 1), lichen planus of the tongue (n = 1), lipoma (n = 3), lymphadenopathy (inflammation, n = 4; metastasis, n = 3; reactive, n = 5), lymphoma (n = 6), oral foul odor (n = 1), oral ulcer (n = 2), otalgia (n = 1), parapharyngeal abscess (n = 1), pharyngolaryngitis (n = 4), salivary gland origin tumor (n = 3), sore throat (n = 3), post-treatment squamous cell carcinoma (n = 13), vallecular cyst (n = 2), and voice change (n = 2).

### CT protocol

**Fig 1:**
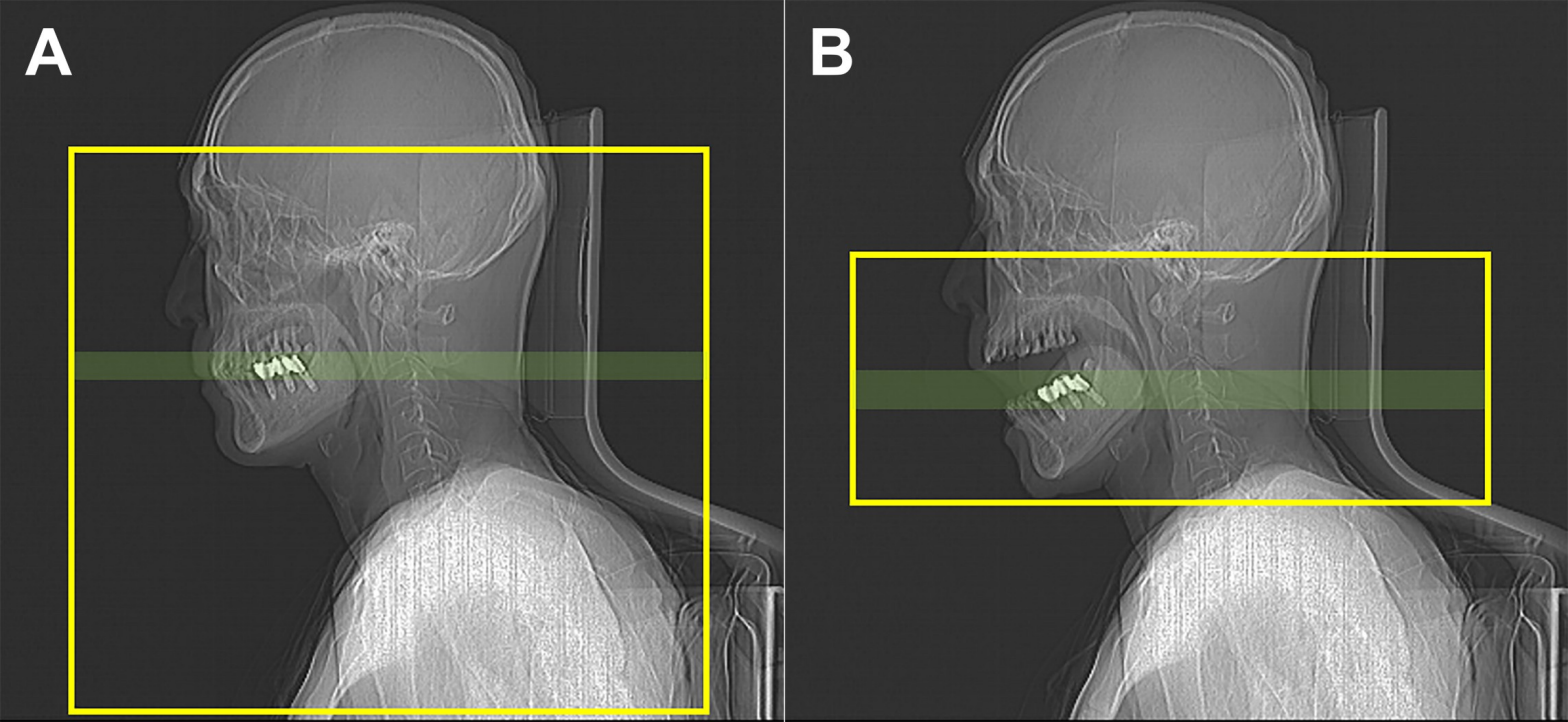
Field-of-view (yellow box) and imaging plane (green box) on lateral scout images of conventional (A) and open mouth (B) computed tomography.

CT images were obtained using 64- (n = 40) and 256-row (n = 19) multidetector scanners (Brilliance, the iQon and the iCT; Philips Healthcare, Best, The Netherlands). A single-phase bolus injection of 100 cc of iodinated contrast media (Iomeron 350 mg/mL, Bracco, UK) was administered at a flow rate of 2 cc/sec. Image acquisition began 80 seconds after the injection, and the scan range was from the frontal sinus to the carina. Other imaging parameters were as follows: 128 × 0.625 collimation, 120 kVp, 250 mAs, 0.835 pitch, and 0.5 rotation time. Images were obtained with a 1-mm slice thickness with 0.5-mm increments. Immediately after obtaining the conventional CT image, an additional open mouth CT scan was performed using the same imaging parameters from the maxillary sinus to the hyoid bone level (Fig 1). During this time, the subjects kept their mouth wide open and breathed quietly through their nose. After scanning, both conventional and open mouth CT images were reconstructed using the O-MAR (Metal Artifact Reduction for Orthopedic Implants, Philips Healthcare) algorithm [3].

### Quantitative image analysis

One radiological technologist (T.E.K. with 8 years of experience with head and neck CT scanning) performed the quantitative imaging analysis. On axial scans of conventional CT (CTc), open mouth CT (CTo), conventional CT with O-MAR processing (CTc_O), and open mouth CT with O-MAR processing (CTo_O) images, circular regions-of-interest (ROIs) were placed on the anatomical subsites of the oral cavity including the mobile tongue, retromolar trigone, and mouth floor, as well as those of the oropharynx including the tongue base, palatine tonsil, and soft palate. The size of each ROI was fixed (400 mm^2^ for the mobile tongue; 70 mm^2^ for the rest of the structures). The reader allocated the ROI in the slice where the anatomical structure was mostly seen. The noise level was defined as the standard deviation of the Hounsfield units in each ROI of the structures. Values were measured twice on the same image, and the averaged two values were used for further analysis.

### Qualitative image analysis

A board-certified neuroradiologist (Y.J.B. with 10 years of experience) and radiological technologist (T.E.K.) independently performed visual inspection of the CTc, CTo, CTc_O, and CTo_O images for the anatomical subsites of the oral cavity and oropharynx. The degree of streaking artifact with the mediastinal window setting (window level and width of 40 and 280 Hounsfield units, respectively) was assessed using a 4-point visual score (Table 1) [3]. The overall subjective image quality was separately examined according to the 5-point visual score (Table 1) [3]. To avoid possible bias, two readers had one-week intervals between the visual assessment of the CTc, CTo, CTc_O, and CTo_O images. The final visual scores for each category were agreed upon in consensus.

**Table 1:** Definition of visual scores.

Degree of streaking artifact		Overall subjective image quality	
Score	Definition	Score	Definition
1	Extensive streak artifact	1	Severe artifact, non-diagnosable image
2	Moderate streak artifact, interfering with the depiction of adjacent structures	2	Poor image quality, partially non-diagnosable
3	Minimal streak artifact, but without interfering with adjacent structures	3	Moderate image quality, limited diagnostic confidence
4	No streak artifact	4	Good image quality, sufficient for diagnosis
		5	Excellent image quality with no artifact

### Statistical analysis

Continuous variables are expressed as the mean ± standard deviation. The noise levels among the CTc, CTo, CTc_O and CTo_O images were compared using a repeated-measures analysis of variance (ANOVA). Mauchly’s test of sphericity was used to evaluate the sphericity assumption, and the Greenhouse-Geissler correction was considered if sphericity was violated. Pairwise comparisons were performed using paired *t*-tests with Bonferroni corrections. The interobserver agreement of the qualitative visual scales between the two readers was tested using the intraclass correlation; greater than or equal to 0.75, excellent agreement; 0.60–0.74, good agreement; 0.40–0.59, fair agreement; and less than 0.40, poor agreement [10]. The visual scores for the streaking artifacts and subjective image qualities among the 4 CT protocols were also compared using repeated measures ANOVAs. Pairwise comparisons were performed using paired *t*-tests with Bonferroni corrections. *P* values less than 0.05 were considered to indicate statistical significance. For pairwise comparisons (with a Bonferroni correction), *P* values less than 0.008 were considered statistically significant. All statistical analyses were performed using SPSS version 25.0 (SPSS, Chicago, IL, USA) and MedCalc 17.9 (MedCalc, Mariakerke, Belgium).

## Results

### Quantitative assessment

**Fig 2:**
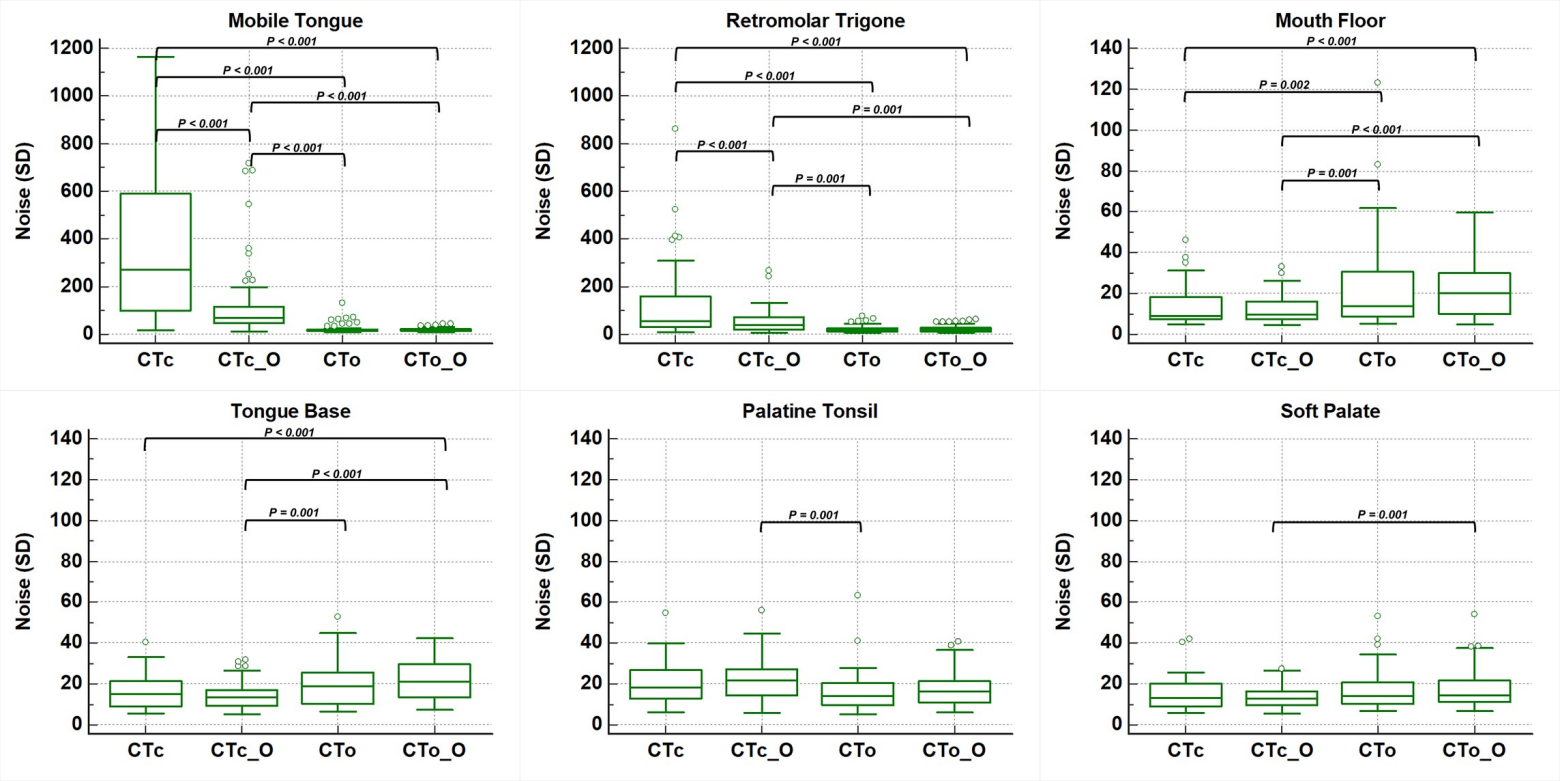
Box-and-whisker plots of the quantified noise level according to the anatomical structures and Computed Tomography (CT) protocols. The bottom and top of box are the first and third quartiles, the horizontal line represents the median value, and the vertical line represents the difference between the maximum and minimum values. Statistically significant differences with *P* values less than 0.008 are presented in the figure. CTc = conventional CT; CTc_O = conventional CT with O-MAR processing; CTo = open mouth CT; CTo_O = open mouth CT with O-MAR processing; O-MAR = metal artifact reduction for orthopedic implants.

The results of the noise measurements for the CTc, CTo, CTc_O, and CTo_O images are shown in Table 2 and Fig 2.

**Table 2:** Quantitative noise measurement.

	CTc	CTc_O	CTo	CTo_O	*P* values
**Oral cavity**					
Mobile tongue	385.47 ± 327.19	128.21 ± 163.64	22.80 ± 20.57	18.40 ± 8.44	< 0.001*
Retromolar trigone	123.82 ± 20.34	53.21 ± 6.74	21.31 ± 2.13	22.18 ± 1.96	< 0.001*
Mouth floor	13.73 ± 9.46	12.21 ± 6.70	22.92 ± 21.31	22.20 ± 13.68	< 0.001*
**Oropharynx**					
Tongue base	15.98 ± 7.77	14.06 ± 6.27	19.22 ± 9.67	21.53 ± 9.54	< 0.001*
Palatine tonsil	20.73 ± 10.59	22.11 ± 9.42	15.79 ± 9.42	17.82 ± 8.51	< 0.001*
Soft palate	14.98 ± 7.57	13.46 ± 5.01	16.72 ± 9.60	18.24 ± 10.16	< 0.001*

Note.—

*P values with statistical significance

Data are presented as the mean ± standard deviation.

The results of pairwise comparison are separately presented in Fig 2.

CTc = conventional CT; CTc_O = conventional CT with O-MAR processing

CTo = open mouth CT; CTo_O = open mouth CT with O-MAR processing

O-MAR = metal artifact reduction for orthopedic implants

For the mobile tongue and retromolar trigone, the noise level was significantly lower with CTo and CTo_O than with CTc or CTc_O (all, *P* < 0.001). However, there was no significant difference between CTo and CTo_O (*P* = 0.103 and 1.0, respectively). For the palatine tonsil, which is an oropharyngeal structure, the noise level was also lower with CTo than CTc_O (*P* = 0.001). However, there were no significant differences between the other CT protocols (all, *P* > 0.008).

The noise level was significantly higher for the mouth floor of the oral cavity and tongue base of the oropharynx with CTo_O than with CTc or CTc_O (all, *P* < 0.001); there was no significance difference between CTo and CTo_O (*P* = 0.116). For the oropharyngeal soft palate, CTo_O showed significantly higher noise than CTc_O (*P* = 0.001). There were no significant differences in noise levels between CTc and CTc_O, or between CTo and CTo_O (*P* = 0.170 and 0.703, respectively).

### Qualitative assessment

**Fig 3:**
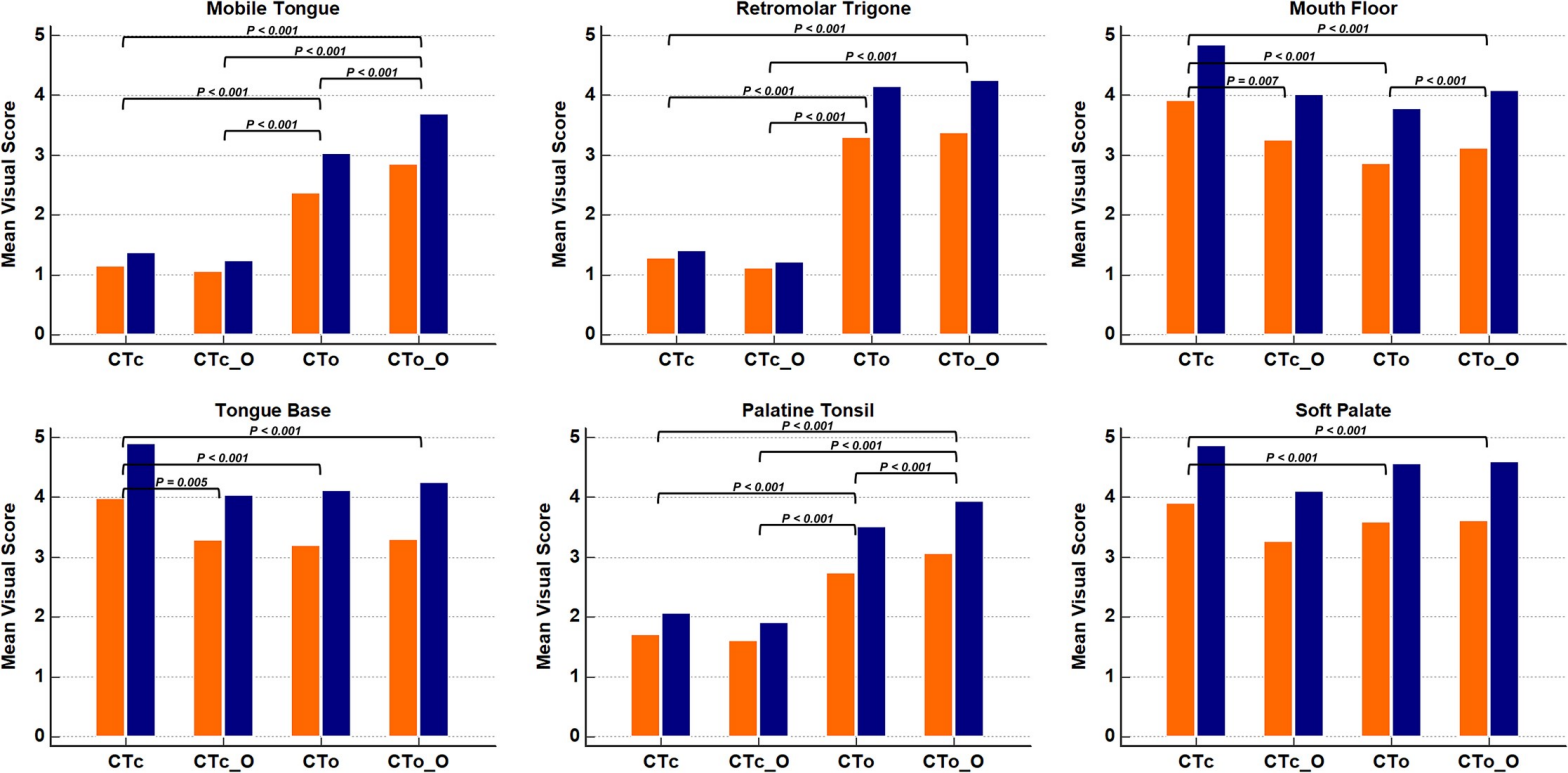
Box graphs of the mean values of the visual scores according to the anatomical structures and Computed Tomography (CT) protocols. The mean values of the 4-point visual scores assessing the degree of streak artifacts are shown in orange box, and the mean values of the 5-point visual scores assessing the overall subjective image quality are shown in the blue box. Statistically significant differences with *P* values less than 0.008 are presented in the figure. CTc = conventional CT; CTc_O = conventional CT with O-MAR processing; CTo = open mouth CT; CTo_O = open mouth CT with O-MAR processing; O-MAR = metal artifact reduction for orthopedic implants.

The interobserver agreement was excellent regarding the 4-point visual scores for the streak artifacts and 5-point visual scores for the overall subjective image qualities (Table 3). Results of the visual scores for CTc, CTo, CTc_O, and CTo_O for each structure are described in Table 4 and Fig 3.

**Table 3:** Intraclass correlation coefficient for visual scores between two readers.

	CTc	CTc_O	CTo	CTo_O
**Streaking artifact**				
** Oral cavity**				
Mobile tongue	0.909 (0.847–0.946)	0.770 (0.590–0.871)	0.924 (0.872–0.945)	0.896 (0.831–0.937)
Retromolar trigone	0.969 (0.948–0.982)	0.968 (0.944–0.982)	0.925 (0.873–0.955)	0.918 (0.863–0.951)
Mouth floor	0.781 (0.632–0.870)	0.777 (0.604–0.874)	0.918 (0.863–0.952)	0.947 (0.911–0.967)
** Oropharynx**				
Tongue base	0.797 (0.659–0.879)	0.793 (0.633–0.883)	0.781 (0.632–0.870)	0.755 (0.588–0.855)
Palatine tonsil	0.915 (0.857–0.949)	0.920 (0.859–0.955)	0.843 (0.736–0.907)	0.848 (0.745–0.910)
Soft palate	0.898 (0.828–0.939)	0.909 (0.839–0.949)	0.960 (0.933–0.976)	0.936 (0.892–0.962)
**Overall subjective quality**				
** Oral cavity**				
Mobile tongue	0.945 (0.907–0.967)	0.887 (0.799–0.937)	0.909 (0.847–0.946)	0.880 (0.800–0.928)
Retromolar trigone	0.987 (0.977–0.992)	0.986 (0.974–0.992)	0.921 (0.868–0.953)	0.932 (0.886–0.960)
Mouth floor	0.930 (0.882–0.958)	0.919 (0.857–0.958)	0.968 (0.946–0.981)	0.963 (0.938–0.978)
** Oropharynx**				
Tongue base	0.852 (0.751–0.912)	0.909 (0.839–0.949)	0.870 (0.782–0.923)	0.88 (0.799–0.929)
Palatine tonsil	0.939 (0.898–0.964)	0.923 (0.864–0.957)	0.867 (0.776–0.921)	0.854 (0.755–0.913)
Soft palate	0.965 (0.941–0.979)	0.934 (0.936–0.980)	0.944 (0.907–0.967)	0.890 (0.815–0.934)

Note.—Data are intraclass correlation (95% confidence interval).

CTc = conventional CT; CTc_O = conventional CT with O-MAR processing

CTo = open mouth CT; CTo_O = open mouth CT with O-MAR processing

O-MAR = metal artifact reduction for orthopedic implants

**Table 4:** Qualitative visual scores.

	CTc	CTc_O	CTo	CTo_O	*P* values
**Streaking artifact**					
** Oral cavity**					
Mobile tongue	1.15 ± 0.41	1.07 ± 0.69	2.37 ± 0.72	2.85 ± 0.55	< 0.001*
Retromolar trigone	1.29 ± 0.74	1.12 ± 0.98	3.31 ± 0.73	3.37 ± 0.67	< 0.001*
Mouth floor	3.92 ± 0.28	3.25 ± 1.50	2.88 ± 0.85	3.12 ± 0.62	< 0.001*
** Oropharynx**					
Tongue base	3.98 ± 0.13	3.29 ± 1.51	3.22 ± 0.89	3.31 ± 0.75	< 0.001*
Palatine tonsil	1.73 ± 0.76	1.61 ± 1.00	2.73 ± 0.69	3.07 ± 0.55	< 0.001*
Soft palate	3.90 ± 0.30	3.29 ± 1.44	3.49 ± 0.56	3.63 ± 0.49	< 0.001*
**Overall subjective quality**					
** Oral cavity**					
Mobile tongue	1.37 ± 0.74	1.24 ± 0.92	3.03 ± 0.98	3.69 ± 0.79	< 0.001*
Retromolar trigone	1.41 ± 0.98	1.22 ± 1.10	4.15 ± 0.89	4.25 ± 0.78	< 0.001*
Mouth floor	4.85 ± 0.36	4.02 ± 1.86	3.78 ± 0.98	4.08 ± 0.73	< 0.001*
** Oropharynx**					
Tongue base	4.90 ± 0.30	4.03 ± 1.87	4.12 ± 1.10	4.25 ± 0.88	< 0.001*
Palatine tonsil	2.07 ± 1.11	1.92 ± 1.33	3.51 ± 0.86	3.93 ± 0.72	< 0.001*
Soft palate	4.86 ± 0.35	4.10 ± 1.79	4.56 ± 0.62	4.59 ± 0.53	< 0.001*

Note.

*P values with statistical significance

Data are presented as the mean ± standard deviation.

The results of pairwise comparison are separately presented in Fig 3.

CTc = conventional CT; CTc_O = conventional CT with O-MAR processing

CTo = open mouth CT; CTo_O = open mouth CT with O-MAR processing

O-MAR = metal artifact reduction for orthopedic implants

**Fig 4:**
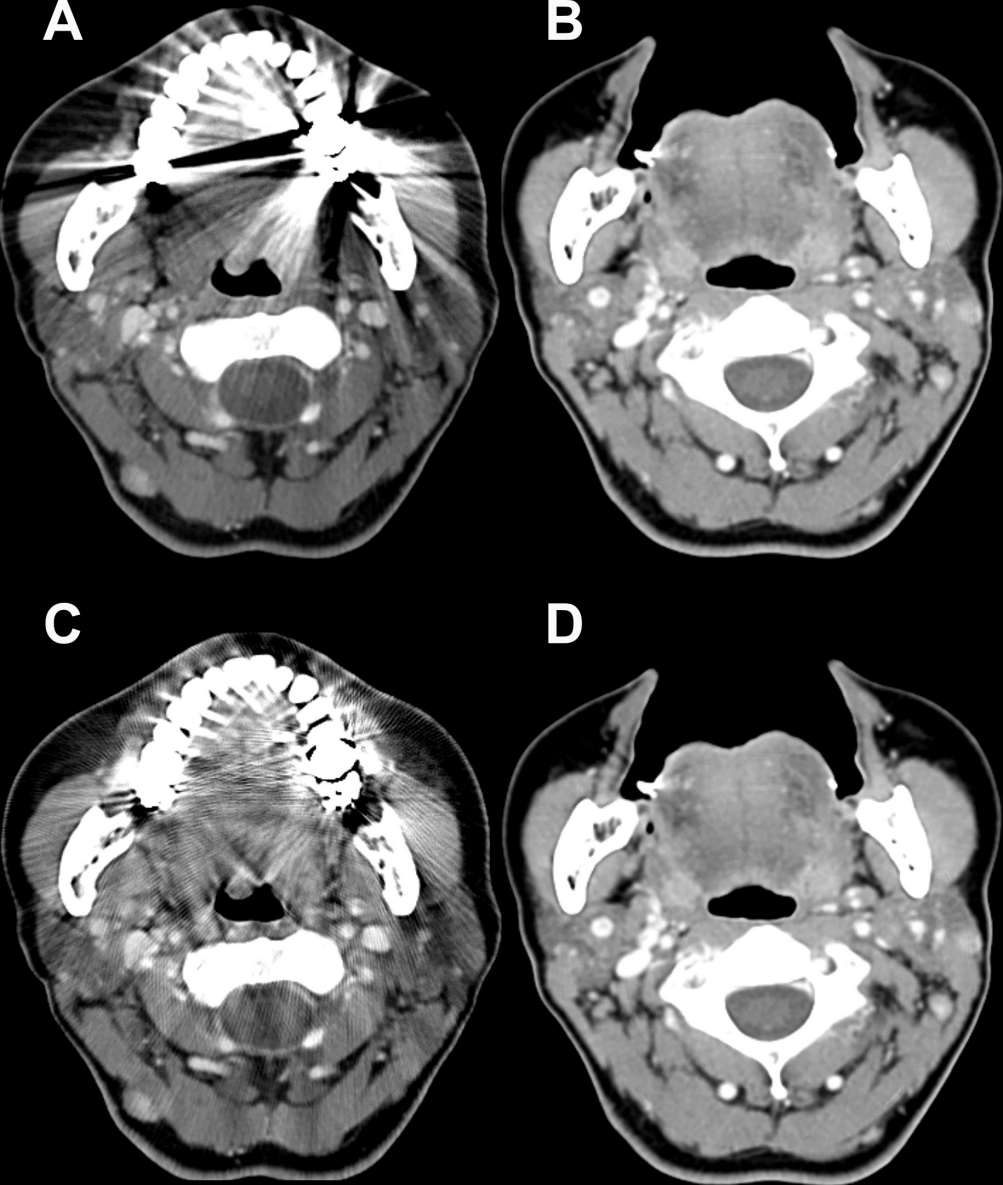
Post-contrast Computed Tomography (CT) images of a 56-year-old woman with a dental prosthesis. On the conventional CT image, the mobile tongue is barely seen due to the metallic artifact (A). However, the structure is clearly seen on the open mouth CT image (B), without overlapping dental prosthesis in the same imaging plane. (C) The additional application of O-MAR to conventional CT shows a substantial decrease in the extent of streak artifacts, but there are still remaining streaks. (D) On open mouth CT images with O-MAR processing, the added effect of O-MAR is not noticeable, since the streak artifact is already displaced from the open mouth maneuver. O-MAR = metal artifact reduction for orthopedic implants.

For the mobile tongue and retromolar trigone, the mean visual scores for the streak artifact and the subjective image quality were significantly higher with CTo and CTo_O than with CTc or CTc_O (all, *P* < 0.001). Only for the mobile tongue, the CTo_O showed significantly higher visual scores than CTo for both categories (all, *P* < 0.001) (Fig 4).

**Fig 5:**
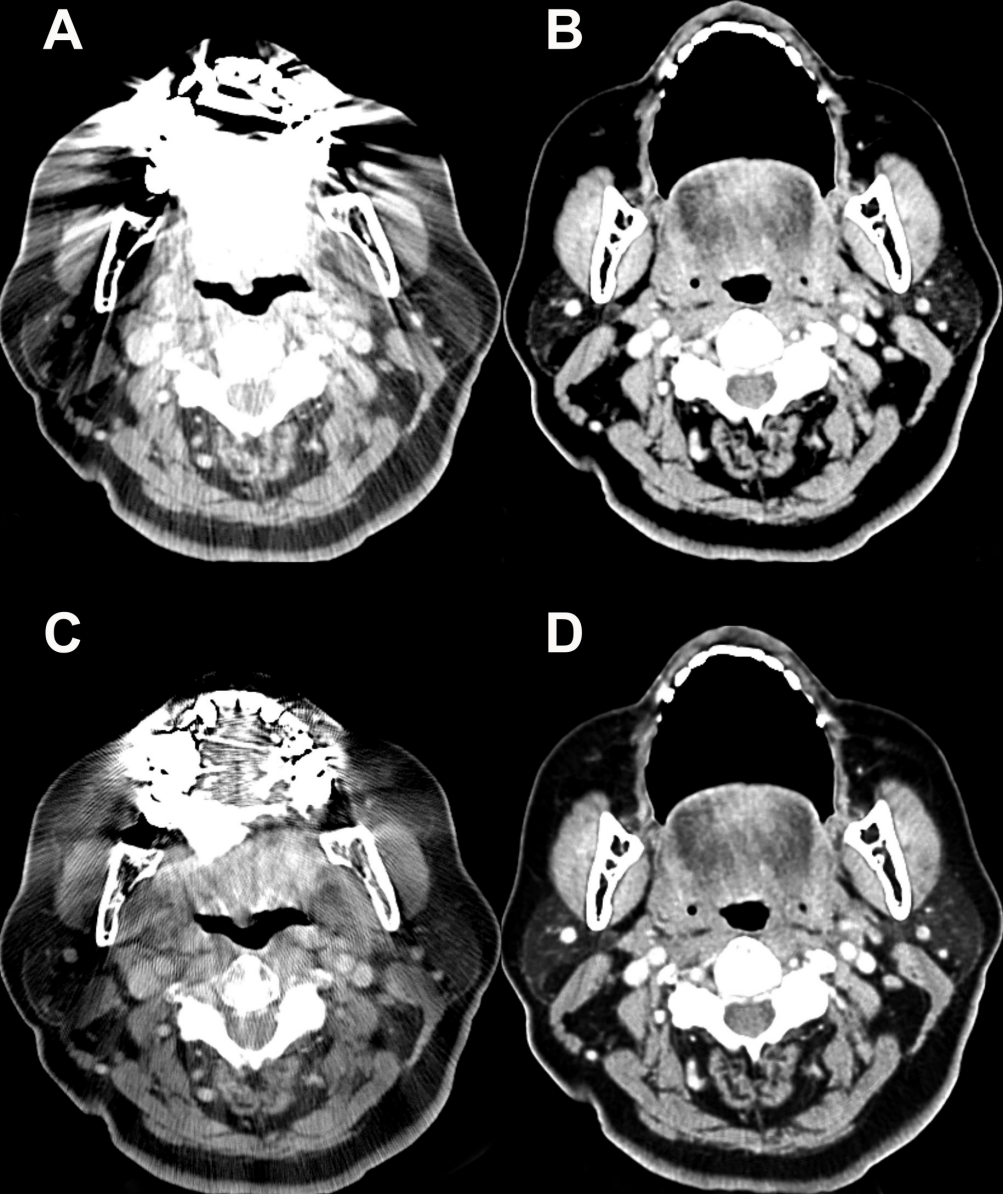
Post-contrast Computed Tomography (CT) images of a 71-year-old woman with a dental prosthesis. Bilateral palatine tonsils are completely covered by the metallic artifact from the adjacent dental prosthesis on a conventional CT image (A). However, on the open mouth CT image (B), there is no streak artifact obscuring the bilateral palatine tonsils, because the axis of the streak artifact from the dental prosthesis is tilted. On a conventional CT image with O-MAR processing (C), the bilateral palatine tonsils are partly seen with decreased artifacts, but the image texture becomes blurry. The added effect of O-MAR is not noticeable on the open mouth CT image with O-MAR processing (D) since the streak artifact is already displaced from the open mouth maneuver. O-MAR = metal artifact reduction for orthopedic implants.

For the palatine tonsil, the mean visual scores for the streak artifact and subjective image quality were also significantly higher with CTo than CTc or CTc_O (all, *P* < 0.001). Furthermore, the mean visual scores increased even higher with CTo_O than with CTo; both categories were statistically significant (all, *P* < 0.001) (Fig 5).

**Fig 6:**
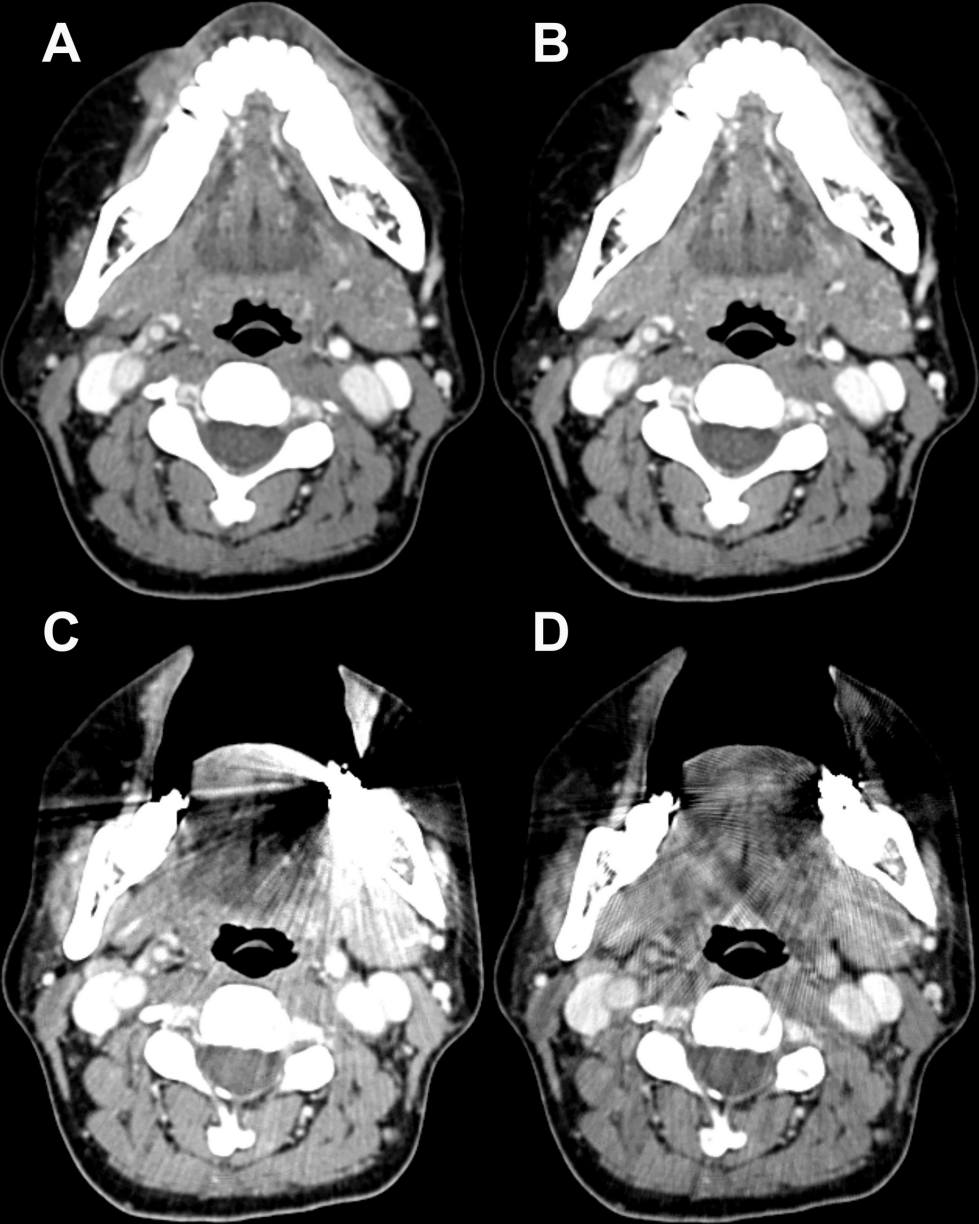
Post-contrast Computed Tomography (CT) images of a 51-year-old woman with a dental prosthesis. On a conventional CT image with the mouth closed (A) and on the conventional CT image with O-MAR processing (B), the mouth floor with extrinsic tongue muscles are well visualized without any artifacts. Contrarily, the structure is degraded by the metallic artifact from the lower molar prosthesis on the open mouth CT image (C). The streak artifact remains substantial even on the open mouth CT image with O-MAR processing (D). O-MAR = metal artifact reduction for orthopedic implants.

In contrast, for the mouth floor, tongue base, and soft palate, the mean visual scores for the streak artifact and subjective image quality were significantly higher with CTc than with CTo or CTo_O (all, *P* < 0.001) (Fig 6). However, for the mouth floor and tongue base, the mean visual scores decreased with CTc_O than CTc for both categories (streak artifact, *P* = 0.007 and 0.005, respectively; subjective image quality, *P* = 0.007 and 0.005, respectively). There were no significant differences in the scores between CTc_O, CTo, or CTo_O (all, *P* > 0.008).

## Discussion

In this study, we comprehensively analyzed the effect of the open mouth maneuver and O-MAR, alone and in combination, on the objective and subjective CT imaging quality of the oral cavity and oropharynx. Our results showed that the quantified noise level, the degree of streak artifact, and the overall subjective image quality significantly improved with open mouth CT imaging compared to conventional CT imaging for the mobile tongue, retromolar trigone, and palatine tonsil, but significantly worsened with the open mouth CT for the mouth floor, tongue base, and soft palate. The additional use of O-MAR significantly improved the degree of streak artifact and the subjective image quality of open mouth CT for the mobile tongue and palatine tonsil.

There have been several maneuvers to reduce beam hardening artifact from dental hardware. It is sometimes possible to avoid artifact by changing patient position or by tilting the gantry [11]; however, changing position can be difficult to apply in some cases such as pediatric patients or those who need immobilization or sedation, and some CT vendors may not provide gantry tilting. Dynamic maneuvers are other options to avoid dental artifact. For instance, to improve the evaluation of the buccal space, dynamic maneuver such as puffed cheek technique can be used [5, 12]; but, the usefulness of this technique is limited to the oral cavity. Among the dynamic maneuvers, the open mouth technique can provide wider indications for increasing visibility of the tumor in the oral cavity and oropharynx [5–7]. Although seemingly effective, only few studies have confirmed the usefulness of the open mouth maneuver in real clinical settings [5, 7]. Recently, in 2019, Bron et al. [7] examined the open mouth procedure and the additional tongue extended maneuver on clinical tumor staging of the oral cavity and oropharyngeal squamous cell carcinoma and showed that these maneuvers could improve the assessment of local tumor extension. However, additional extension of the tongue would likely cause a significant amount of inconvenience to patients and additional motion artifacts. Another limitation of this study was that the authors did not consider the detailed anatomical structures of the oral cavity and oropharynx. According to the 8^th^ edition of the American Joint Committee on Cancer staging system [13], the topography of the oral cavity includes the tongue, gums, mouth floor, hard palate, and retromolar area, and that of the oropharynx includes the tongue base, tonsil, soft palate, and vallecula. Since each subsite is located at a different level of the head and neck with reference to the dental prosthesis, consideration should be given regarding the anatomical structures as well as a separate analysis of the image quality for different subsites.

Another method to overcome the metallic artifact in the head and neck is a post-processing metal artifact reduction algorithm such as O-MAR [1, 3, 4, 8, 9]. O-MAR is a commercial product that implements iterative reconstruction to mitigate metallic artifacts on CT images [9, 14]. Many studies have shown that the degree of the depiction of the relevant structure is improved with the O-MAR image compared to the non-O-MAR image of the head and neck, reducing metallic artifacts, while maintaining a clinically acceptable processing time [1, 3, 9, 14]. Additionally, in previous studies that performed qualitative image assessment, O-MAR reduced streak artifacts, thereby enhancing the overall image quality of the CT image, compared to the non-O-MAR image [1, 3]. However, O-MAR cannot completely eliminate metallic artifacts; it can only enable the depiction of the structures, and its clinical application is still limited in settings with multiple metallic objects, such as dental fillings [3, 9, 14]. In addition, application of O-MAR results in blurring and an unnatural texture of the image due to the loss of spatial resolution, as well as the discarded metal projection data. Together, these technical challenges prevent O-MAR from routine use in clinical settings for head and neck tumor staging [3].

In our study, we first evaluated the effect of O-MAR and the open mouth maneuver on the CT image quality for the oral cavity and oropharynx, considering the detailed anatomical structures that are included in the 8^th^ edition of the American Joint Committee on Cancer staging system. Our results showed that application of the open mouth maneuver and the O-MAR technique had a different impact on the image quality based on the anatomical subsites of the oral cavity and oropharynx, which has never been reported.

First, among the structures of the oral cavity, the mobile tongue and the retromolar trigone area showed significantly lessened noise on the open mouth view compared to the closed mouth view. We assume that the tilted angle of the dental prosthesis enabled the reduction of the noise in the oral cavity. Additional application of O-MAR did not affect the quantitative noise level, but the degree of streak artifact and the overall subjective image quality were significantly improved for the mobile tongue. We speculate that the reason O-MAR could not redeem the CT image quality for the retromolar trigone area was likely due to limitation of O-MAR application in locations where the metal artifacts were too extensive. To summarize, the open mouth maneuver could benefit the CT images for the mobile tongue and retromolar trigone areas, and addition of the O-MAR technique could improve the image quality for the mobile tongue. However, regarding the mouth floor, the open mouth view caused a significantly higher noise level than the conventional CT. Accordingly, the visual scores for the quality assessments became worse with the open mouth view. We believe that this is likely due to the unintended intrusion of the streak artifact from the tilting angle of the mouth floor. Additionally, application of O-MAR to conventional CT imaging did not affect the noise level, but rather decreased the visual scores, likely due to blurring and the unfamiliar change in the image texture. Thus, although the mouth floor is a subsite of the oral cavity, evaluation of the mouth floor should be based on conventional CT imaging without using the open mouth maneuver.

Second, as for the oropharyngeal structures, scanning of the palatine tonsil could benefit from the open mouth maneuver due to decreased noise. Similar to the mobile tongue and retromolar trigone areas, this finding could be explained by displacement of the streaking artifact by the mouth opening and tilted dental arch. Accordingly, the degree of streak artifact and the subjective image quality improved with open mouth CT. More importantly, the visual scores increased when O-MAR was used in conjunction with open mouth CT. Thus, the best CT approach for the palatine tonsil would be the open mouth CT with O-MAR application. Contrarily, the tongue base and soft palate did not benefit from the open mouth maneuver; the quantitative noise and degree of streak artifact increased with open mouth CT. Application of the O-MAR technique did not improve quantitative noise, and also deteriorated the subjective image quality, likely due to blurring and degraded texture. We assume that this can be explained by the same reason for the increased noise in the mouth floor when opening the mouth, specifically that the structures located above or below the level of the dental arch induce the unintended intrusion of the streak artifact. Consequently, we suggest that CT imaging for the oral cavity and oropharyngeal structures located above or below the dental arch should be scanned with the closed mouth view.

Our study has some limitations. First, we could only include subjects with post-treatment squamous cell carcinomas in the oral cavity and oropharynx, and we could not include subjects with purpose of initial cancer work-up. Thus, we could not assess the degree of tumor depiction or the diagnostic performance of CT imaging for clinical tumor staging. Future studies regarding the clinical use of these CT protocols should be performed to evaluate the diagnostic values. However, since we performed the image analysis based on the normal anatomical structures with CT imaging, we believe that further validation of the utility of the open mouth maneuver and the O-MAR technique in clinical tumor staging can be started from our study. Second, we used a CT system from a single vendor for O-MAR processing; different methods of MAR are provided according to different CT vendors. To apply our findings more widely in clinical settings, different MAR techniques from different vendors should be assessed. Third, two readers could not be blinded to the CT techniques when assessing visual scores for streaking artifact and overall image quality. This could be acted as a bias in the qualitative assessment. Lastly, ROI allocation for the quantitative noise measurement was performed by a single reader. However, the reader aimed to be consistent in placing the ROIs with a fixed area; moreover, the averaged values from the two separate measurements were used in the further analysis. Therefore, we believe that this has little influence on the results.

## Conclusion

In conclusion, application of the open mouth maneuver and O-MAR can have different influences on the CT image quality according to the anatomical subsites of the oral cavity and oropharynx. The open mouth maneuver could provide the best image quality with minimal artifacts for the mobile tongue, retromolar trigone, and palatine tonsil, and the added O-MAR technique could further improve image quality for the mobile tongue and palatine tonsil areas. Contrarily, the CT image quality of the mouth floor and tongue base could be superior when closing the mouth, which might not benefit from the addition of O-MAR.
